# Processing complexity increases in superficial layers of human primary auditory cortex

**DOI:** 10.1038/s41598-019-41965-w

**Published:** 2019-04-02

**Authors:** Michelle Moerel, Federico De Martino, Kâmil Uğurbil, Essa Yacoub, Elia Formisano

**Affiliations:** 1 Maastricht Centre for Systems Biology, Maastricht University, Universiteitssingel 60, 6229 ER Maastricht, The Netherlands; 2 Department of Cognitive Neuroscience, Faculty of Psychology and Neuroscience, Maastricht University, Oxfordlaan 55, 6229 EV Maastricht, The Netherlands; 3 Maastricht Brain Imaging Center (MBIC), Oxfordlaan 55, 6229 EV Maastricht, The Netherlands; 4 Center for Magnetic Resonance Research, Department of Radiology, University of Minnesota, 2021 6th Street SE, Minneapolis, MN 55455 USA

## Abstract

The layers of the neocortex each have a unique anatomical connectivity and functional role. Their exploration in the human brain, however, has been severely restricted by the limited spatial resolution of non-invasive measurement techniques. Here, we exploit the sensitivity and specificity of ultra-high field fMRI at 7 Tesla to investigate responses to natural sounds at deep, middle, and superficial cortical depths of the human auditory cortex. Specifically, we compare the performance of computational models that represent different hypotheses on sound processing inside and outside the primary auditory cortex (PAC). We observe that while BOLD responses in deep and middle PAC layers are equally well represented by a simple frequency model and a more complex spectrotemporal modulation model, responses in superficial PAC are better represented by the more complex model. This indicates an increase in processing complexity in superficial PAC, which remains present throughout cortical depths in the non-primary auditory cortex. These results suggest that a relevant transformation in sound processing takes place between the thalamo-recipient middle PAC layers and superficial PAC. This transformation may be a first computational step towards sound abstraction and perception, serving to form an increasingly more complex representation of the physical input.

## Introduction

The human neocortex consists of six layers, each with a distinct anatomical connectivity and functionality. Across the primary sensory cortices (i.e., primary visual, auditory, and somatosensory cortex) the anatomical build up and connectivity of these cortical layers is relatively similar. Input from the thalamus to the primary sensory cortex arrives in layer IV, cortico-cortical connections arise from superficial layers II-III, and efferent subcortical projections originate from deep layers V–VI^1,2^. As a result of this layer-specific connectivity, the deep, middle, and superficial layers support different functions. While sensory processing in the middle input layers may be relatively simple, inherited from thalamic processing, superficial and deep layers may comprise increasingly complex stimulus representations as a result of columnar processing, horizontal connections, and cortico-cortical feedback^3,4^.

In the primary visual and somatosensory cortex, laminar processing differences have been repeatedly shown. For both sensory modalities, processing is simplest and most representative of the physical sensory input in the thalamo-recipient middle layers, and increases in complexity towards superficial and deep layers^5–7^. For the auditory cortex, consistent principles of laminar processing have been more difficult to demonstrate. While early studies reported conflicting findings^3^, recent studies provide evidence for an auditory laminar organization that is similar to the other early sensory cortices. In the PAC, the fastest responses to sounds are seen in layer IV^8^, followed by deep and superficial layers (but see also reports of faster sound responses in deep layers, possibly due to direct thalamic connections^9,10^). Results from cat PAC showed systematic processing variations throughout the cortical depth, where processing complexity increased with distance from granular layer IV^10^. Accordingly, recordings in the awake marmoset monkey showed that while neurons in middle thalamo-recipient layers respond well to simple sounds, many neurons in superficial PAC could not be that easily driven^11^. Instead, superficial PAC neurons displayed sustained responses to complex sounds and con-specific vocalizations^12^. Thus, while layer IV may contain a relatively faithful representation of the spectrogram of incoming sounds, other cortical depths may incorporate more complex sound representations.

To date, the spatial resolution of non-invasive measurements has prohibited investigating if similar differences in laminar processing are present in the human auditory cortex. This is a crucial omission, as human audition is unique in its flexibility and richness, e.g., for processing speech and language. Therefore, results from animal studies are not directly transferable to the human. Moreover, while the PAC was traditionally considered to be a simple feature analyser, more recent studies highlight its role in processes beyond the physical sound analysis. For example, processing in the PAC is rapidly modulated with changing task demands^13,14^, and is affected by signals from other sensory cortices^15^. Furthermore, the PAC has been implicated in auditory object formation^16^, and auditory category learning^17,18^. These complex auditory processes, going beyond the physical sound analysis, may occur within PAC as information flows through the layers of the canonical microcircuit. Exploring layer-dependent sound processing may shed light on how PAC computations support auditory cognition.

Ultra-high field magnetic resonance imaging (MRI) profits from an increased sensitivity and specificity to neuronal activity, and allows investigating the human brain at a sub-millimetre spatial resolution^19^. Given that the thickness of the human PAC is ~2–3 mm^20^, this sub-millimetre spatial resolution enables acquiring independent signals from deep, middle (presumably reflecting thalamo-recipient layer IV), and superficial cortical depths. Here we investigate responses to natural sounds across cortical depths of the human auditory cortex. We observe that while BOLD responses in deep and middle PAC layers are equally well represented by a simple *frequency* model and a more complex frequency-specific *spectrotemporal modulation* model, responses in superficial PAC are significantly better represented by the more complex model. This suggests that the neuronal populations underlying the responses to natural sounds in superficial PAC display an increase in processing complexity compared to deep and middle PAC layers. This increased processing complexity is present throughout cortical depths in the non-primary auditory cortex as well. Thus, a transformation in sound processing takes place between middle and superficial PAC. This transformation may be a key step from a representation of the physical sensory input toward an increasingly complex, categorical, and abstract sound representation.

## Results

We obtained high resolution anatomical data (0.6 mm isotropic) and functional data (0.8 mm isotropic) while subjects listened to natural sounds. We observed significant responses to the sounds throughout the supratemporal plane, including HG (the putative location of primary auditory cortex), Heschl’s sulcus, planum polare (PP), planum temporale (PT), and the parts of the superior temporal gyrus and sulcus that were covered by the fMRI field of view.

**Figure 1:**
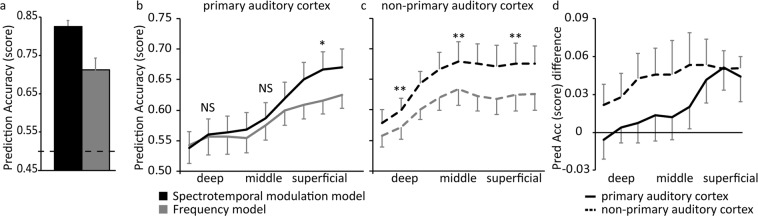
Model performance. Model performance is evaluated by the prediction accuracy of responses to test sounds (i.e., sound identification score). (**a**) Performance of the models on the entire dataset, which covers the majority of the supratemporal plane (STP) and parts of the superior temporal gyrus (STG). The dashed line corresponds to chance performance (score = 0.5), and the error bars indicate the standard error across subjects (*N* = 6). (**b**,**c**) The cortical depth dependent model performance ranging across deep, middle, and superficial grey matter for the part of the grid with the highest (i.e., primary auditory cortex) and lowest (i.e., non-primary auditory cortex) 50% of myelin-related contrast in solid and dashed lines, respectively. Statistical results are indicated by NS (not significant), *p < 0.05, and **p < 0.001. (**d**) The cortical depth dependent difference in model performance (ranging across deep, middle, and superficial grey matter) between primary auditory cortex and non-primary auditory cortex. In b-d, the error bars indicate the standard error across hemispheres (*N* = 12).

Cortical responses to the sounds were analyzed with two encoding models, representing different hypotheses on sound processing. A first *frequency* model was simplest, describing sound processing in terms of the frequency preference of cortical neuronal populations. A second *spectrotemporal modulation* model described cortical sound processing as the frequency-specific tuning of neuronal populations to combined spectral and temporal modulations. The performance of the models based on the responses in the auditory cortex as a whole (including all voxels with a significant response to the sounds; *p* < 0.05 uncorrected) is shown in Fig. 1a. Both the simpler *frequency* model and the *spectrotemporal modulation* model could predict responses to testing sounds above chance (average prediction accuracy [SEM] = 0.83 [0.02] for the *spectrotemporal modulation* model, and 0.71 [0.03] for the frequency model), and thus provided a meaningful representation of sound processing in the auditory cortex. In agreement with our previous study^21^, the *spectrotemporal modulation* model outperformed the *frequency* model (two-sided paired t-test after Fisher transformation of the prediction accuracy values; t(5) = 7.19; *p* = 8.09 × 10^−4^). This finding was not driven by a subset of sounds, but instead was observed across the large majority of sound categories (Fig. S1a).

**Figure 2:**
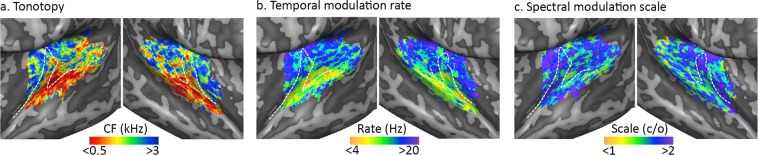
Group topographic maps. (**a**–**c**) CBA-based group maps for preferred frequency (tonotopy), temporal modulation rate, and spectral modulation scale, on an inflated representation of the left and right hemisphere (in the left and right column, respectively). The white dashed line outlines HG.

We further examined the fitted *spectrotemporal modulation* model by creating large-scale topographic maps for each of the three acoustic features. Maps of tonotopy (i.e., best frequency [BF]), preferred temporal modulation rate, and preferred spectral modulation scale are shown in Fig. 2. The tonotopy maps were in accordance with previous reports^22–25^. That is, the tonotopy maps showed a cortical region responding best to low frequencies situated on the posterior part of HG and anterior part of Heschl’s sulcus, surrounded anteriorly and posteriorly by regions preferring higher frequencies (Fig. 2a). The temporal modulation rate and spectral modulation scale maps displayed both similarities and differences compared to previous findings. We observed a significantly negative correlation between the temporal modulation rate and spectral modulation scale map (mean [SE] = −0.027 [0.002]; *p* = 0.031; two-tailed non-parametric signed rank test on Fisher transformed correlation values between individual maps prior to spatial smoothing) in agreement with previous results^21,26^, yet this correlation was weaker than reported before^21^. Consistent with our previous results^21^, the medial and lateral part of the STP preferred higher and lower temporal modulation rates, respectively (Fig. 2b). More specifically, the cortical region at the posterior adjacency of the medial HG preferred the highest modulation rates, while regions along the STG preferred the lowest temporal modulation rates. In the maps of spectral modulation scale preference, observations consistent with our previous results include tuning to higher spectral modulation scales along the anterior part of HG and on the PP, and a preference for lower spectral modulation scales in the majority of the PT and STG (Fig. 2c).

**Figure 3:**
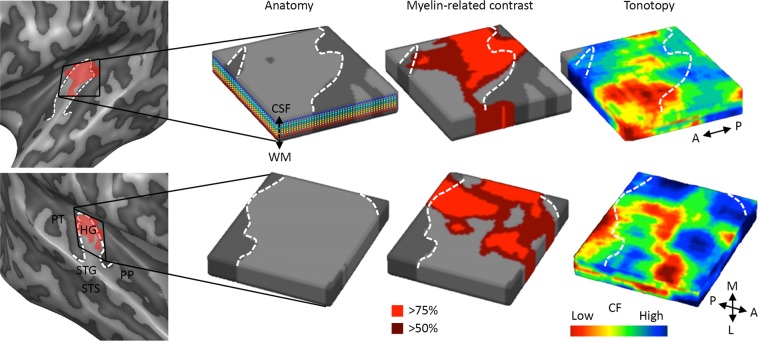
Analysis pipeline. The left and right temporal lobe (top and bottom row, respectively) of a representative subject (S2; see Supplementary Fig. 2 for the data of all individuals). Light and dark grey regions represent gyri and sulci respectively. The white dashed line outlines Heschl’s gyrus (HG). Major anatomical landmarks are shown in (**b**; first column), and include HG, planum polare (PP), planum temporale (PP), the superior temporal gyrus (STG) and the superior temporal sulcus (STS). The red shaded region in the first column approximates the region with highest myelin-related contrast (MRC; >50%). For each subject and hemisphere a cortical depth-dependent grid, situated on the postero-medial part of HG, is defined. The cortical ribbon is sampled at nine cortical depths, range from approaching the white matter (WM) to approaching the cerebrospinal fluid (CSF; coloured lines in second column of **a**). The cortical depth dependent grids are used to sample the MRC maps, which are thresholded to define the primary auditory cortex (PAC; MRC > 50%, bright and dark red colours in the third column), and non-PAC (MRC < 50%; light and dark grey colours in the third column). The cortical depth dependent grids are furthermore used to sample the functional data, allowing for cortical depth dependent fitting of the computational models and resulting in cortical depth dependent maps of feature preference (e.g., tonotopy; fourth column).

Next, we explored the performance of the sound representation models at a smaller spatial scale. That is, as the primary auditory cortex (PAC) is more densely myelinated than surrounding auditory regions^27,28^, we used myelin-related contrast (MRC) maps to obtain a non-invasive estimate of the PAC in each hemisphere (Fig. 3). Throughout hemispheres we localized PAC to the postero-medial part of HG (Figs 3 and S2). The region identified as PAC moved posteriorly when incomplete or complete duplications of HG were present (e.g., the left hemisphere of S1 in Fig. S2). Our definition of PAC covered approximately half of the HG.

Finally, we analysed model performance in the PAC and the non-PAC, at deep, middle, and superficial cortical depth (Fig. 1b–d). The GE-EPI signal is strongest at the cortical surface, and this has been shown to correlate with an overall prediction accuracy increase towards superficial cortical depths^29^. The improved performance of both models towards the cortical surface, as can be observed in Fig. 1b,c, should therefore not be interpreted as a neuroscientific result. On the other hand, we previously confirmed experimentally that the cortical depth dependent *difference* in model performance removes the effect of the signal strength (and thus the bias towards the surface)^29^, making the depth dependent effects interpretable (Fig. 1d). In the PAC only, we observed a significant interaction such that the *difference* in model prediction accuracy varied with cortical depth (two-way RM ANOVA after Fisher transformation of the prediction accuracy values; significant interaction between the factors ‘Computational Model’ and ‘Cortical Depth’; F[2,22] = 5.31; *p* = 1.31 × 10^−3^). Only at a superficial depth of the PAC, did the *spectrotemporal modulation* model significantly outperform the *frequency* model (one-sided paired t-test; t(11) = 3.01; *p*(corrected) = 1.79 × 10^−2^). In the non-PAC, there was a significant difference in model performance (main effect of ‘Computational Model’; F[1,11] = 7.12; *p* = 2.18 × 10^−2^) which did not vary with cortical depth (no significant interaction between the factors ‘Computational Model’ and ‘Cortical Depth’; main effect of ‘Cortical Depth; F[2,22] = 5.77; *p* = 9.60 × 10^−3^). In other words, the *spectrotemporal modulation* model significantly outperformed the frequency model in these non-primary auditory regions uniformly throughout cortical depth. The improved performance of the *spectrotemporal modulation* model in the non-PAC compared to the PAC was not confined to a subset of sounds, but was observed for all sound categories (Fig. S1b).

## Discussion

To date, it remains unclear if the laminar anatomical connectivity pattern of the human PAC is paralleled by layer-specific variations in sound processing. Using ultra-high field MRI, we observed that BOLD responses in middle PAC layers were equally well represented by a simple *frequency* model and the *spectrotemporal modulation* model. Instead, BOLD responses in superficial PAC were significantly better represented by the *spectrotemporal modulation* model, suggesting that neuronal populations in superficial PAC underlying the BOLD responses displayed an increased processing complexity. This increased complexity of sound processing was propagated throughout cortical depths in non-primary auditory cortex, and in accordance with our previous report at lower field strength and spatial resolution^21^, was preserved throughout the supratemporal plane. The observed increase in processing complexity in superficial PAC and throughout non-PAC may reflect neuronal sensitivity to acoustic feature combinations, such as previously described in the auditory cortices of echo-locating bats^30^, songbirds^31^, and the marmoset monkey^12^. Tuning to acoustic feature combinations may serve as a stepping stone towards the creation of an auditory object, and provide a mechanism by which those sounds that are ecologically important can be more efficiently (i.e., sparsely) represented^12^. Overall, the modified sound representation in superficial PAC may be a first computational step in the transformation of the physical input towards sound abstraction and perception^32^.

Sound processing complexity in superficial PAC may increase through processes operating at a local spatial scale, resulting from the flow of information throughout the canonical microcircuit^3^. Previous electrophysiological studies showed that processing of spectral and temporal modulation information undergoes a radical change from thalamus to the granular PAC input layers^33^ and, within PAC, with increased distance from middle input layers^10^ or in superficial layers^12^. Our results show an increase in processing complexity when moving from middle to superficial cortical depths. However, we did not observe an increased processing complexity in infragranular layers, as may have been expected based on cat recordings^10^ and laminar processing principles in other early sensory cortices^5,7^. This discrepancy could represent a species-specific difference. While processing complexity increases in infragranular cat PAC, this may not be present in the marmoset monkey^12^ or in the human. An exclusive change in supragranular PAC processing complexity may result through influences originating within the auditory cortex, through lateral connections of pyramidal cells in superficial PAC layers^6^ or as a result of short-distance feedback projections from secondary auditory cortical areas^34^. Alternatively, the reason for not observing increased processing complexity in infragranular PAC could be methodological. BOLD fMRI is an indirect measure of neuronal activity, which represents neuronal input (i.e., LFP) rather than spiking activity^35^. Input to middle PAC is thalamic, and also input to deep PAC layers may be partially thalamic^9^. The thalamic input to deep and middle layers could have driven the BOLD responses that we obtained from these cortical depths, while the BOLD responses collected from superficial cortical depths may have reflected cortical auditory processing^33^.

Two methodological issues related to the execution of the analysis and the interpretation of our findings require further discussion. First, the non-invasive identification of the PAC is still a matter of debate, largely resulting from the fact that tonotopic maps alone are insufficient to delineate the PAC^36^. Non-invasive functional measures beyond tonotopy have been proposed to identify the PAC^37–39^, but none of these have been consistently adopted. The PAC is more densely myelinated than surrounding auditory regions^27,28^ and, consequently, maps of myelin related contrast (MRC) were shown to be valuable for its localization^40–42^. Myelination and, correspondingly, the MRI-based MRC, varies gradually throughout the STP. We considered the half of the grid with the highest MRC as the PAC. While this threshold is necessarily arbitrary, it resulted in plausible PAC definitions. That is, the localization of the PAC to the postero-medial part of HG, as well as the posterior movement of PAC with (in)complete HG duplications, is consistent with results from cytoarchitecture^27,43–45^. While the size of our PAC, covering approximately half of the entire HG, is a smaller cortical area than the definition in a number of previous fMRI studies^23,46^, this size concurs with cytoarchitectonic PAC definitions^47^. Overall, while it is possible that our definition of PAC did in fact not cover the complete PAC, it is highly likely that the region we refer to as PAC is indeed fully situated within the primary auditory cortex. The remainder of the grid, which we refer to as non-PAC, covers regions anterior and posterior to HG. In most hemispheres, it included the anterior part of HG and the first transverse sulcus, and posteriorly extended to Heschl’s sulcus, the lateral part of a second HG when applicable, and the anterior part of the PT. These regions can be considered secondary auditory cortex (medial and lateral belt respectively), and may reflect part of regions ‘MBelt’ and ‘LBelt’ as recently described by Glasser *et al*.^48^.

The second methodological issue that requires additional discussion concerns the spatial specificity of our fMRI measurements. Using GE-EPI, the acquired fMRI signal contains contributions from large pial veins situated on top of the grey matter^49^, diving veins penetrating the grey matter, and the microvasculature^50^. As a result of the pial vein contribution, the observed signal changes are strongest at the cortical surface (high sensitivity) and spatially less specific than those at deeper cortical depths (low specificity)^51–53^. A first implication of these cortical depth-dependent changes in sensitivity and specificity is that GE-EPI cortical depth-dependent topographic maps may be biased in the assignment of the voxels’ preferred feature^29,51,52^. We therefore do not report cortical depth-dependent maps of tonotopy and spectrotemporal modulation preference. These cortical depth-dependent maps were previously reported based on a more spatially specific, predominantly T_2_-weighted, dataset^54^. A second implication is that an alternative explanation for the difference in model performance between PAC and non-PAC observed in our data may exist, namely that the higher performance of the *spectrotemporal modulation* model in superficial PAC is merely a vascular artefact. For example, the higher *spectrotemporal modulation* model performance may have ‘bled in’ from non-PAC, and did so only in superficial layers as this cortical depth has the lowest spatial specificity due to its proximity to pial veins. While it is difficult to exclude this scenario altogether, the results reported in our previous study make this alternative explanation unlikely^29^. If the increased performance of the *spectrotemporal modulation* model compared to the *frequency* model in superficial PAC was a large vein artefact, we would expect this relative increase in model performance to be strongest in superficial locations *closest* to veins. Instead, after quantification of each gridpoint’s distance to a vein we observed that while the overall model performance was dependent on proximity to veins, the *difference* in model performance was *unrelated* to vein proximity (see Fig. 5f in^29^). Thus, in spite of the contribution of large veins to the signal, we argue that through model comparison and careful evaluation of the data, the cortical depth dependent effect is reliable and interpretable.

Feature-selective auditory attention, auditory category learning, and input from other sensory modalities have been shown to modify primary auditory processing^14,15,18,55^. While our current results characterized fundamental processing differences across the layers of the human primary auditory cortex, the major functions of the laminar architecture of the neocortex may only emerge fully when it is engaged in such behaviourally relevant tasks. Our results, describing stimulus-evoked laminar PAC processing, provide a framework and the methodology that can be employed for exploring how laminar processing differences in PAC support auditory behaviour throughout increasingly complex environmental settings and demands.

## Methods

### Ethics statement

The Institutional Review Board (IRB) for human subject research at the University of Minnesota approved the experimental procedures. The experiment was performed in accordance with the approved guidelines and the Declaration of Helsinki. We obtained written informed consent from each participant before the measurements were started.

### Participants

The participants consisted of six healthy volunteers (four females and two males; mean age [SD] = 28.5 [7.8]) who did not have a history of neurological disease or hearing disorders.

### Experimental stimuli and design

We described the data analysed in this study in an earlier methodological study^29^ that examined how a T_2_- vs. a T_2_*-weighted dataset, both acquired at submillimetre spatial resolution, performed across various fMRI analyses. In that study we observed that the T_2_-weighted data was preferable for the examination of cortical depth dependent preference maps requiring high specificity, while the T_2_*-weighted data was preferable for model-based comparisons. Therefore, we now compare the performance of computational models based on an optimized subset of the dataset. The anatomical data reported in this study was previously used in a study that examined the columnar stability of preference maps in auditory cortex^54^.

In the current study we used the data of the first two of the original three scanning sessions. In the first session, we collected high resolution anatomical data for segmenting the white matter (WM) – grey matter (GM) boundary and GM – cerebrospinal fluid (CSF) boundary. We furthermore used the anatomical data to sample the cortical layers^56^ and to compute a myelin related contrast (MRC) map^41,42^. In the second session, we collected GE-EPI data at high spatial resolution while complex, natural sounds were presented to the participants. We presented a total of 144 sounds that covered six categories: speech utterances, voice sounds, animal cries, musical instruments, tool sounds, and scenes of nature (24 sounds per category). The sampling rate of the sounds was 16 kHz and they were 1000 ms in duration. We equalized the energy (RMS) across sounds, and ramped their onset and offset with a 10 ms linear slope. We used MRI-compatible S14 model earbuds of Sensimetrics Corporation (www.sens.com) to present the sounds in the scanner.

We divided the 144 sounds into 4 non-overlapping sets (36 sounds per set). This division was pseudorandom as we ensured that each set contained an equal number of sounds of each semantic category (i.e., 6 category exemplars per set). Data in the second scanning session was collected in 12 fMRI runs. We presented one set of 36 sounds per run, and each sound was repeated once in that run. A set of sounds was presented in three runs, such that each sound was presented a total of three times across the whole experiment. We jittered the inter-stimulus interval with 2, 3, or 4 TRs, and played the sounds with an additional random jitter in the silent gaps between the functional data acquisition. We also added 8% of silent trials to increase the variability in the acquired BOLD signal. Participants performed a one-back task, and pressed a button if they heard the same sound in two consecutive trials. This occurred in 6% of the trials, and we excluded these “repeat trials” from the analysis.

### Acquisition of MRI data

We performed the measurements using a 7 Tesla whole body magnet (Magnex Scientific, Abingdon, UK) that was driven by a Siemens console (Siemens Medical Systems, Erlangen). Specifically, a 32 channel loop transceiver (custom-built, whole head) along with a high performance head gradient insert was used. In the first session we collected the following anatomical datasets: Two T_1_ datasets, a proton density [PD] dataset, and a T_2_* weighted dataset at a high spatial resolution (voxel size = 0.6 mm isotropic). The T_1_ weighted data was acquired using a modified magnetization-prepared rapid gradient-echo (MPRAGE) sequence (repetition time [TR] = 3100 ms; time to inversion [TI] = 1500 ms; time echo [TE] = 3.45 ms; flip angle = 4°; generalized autocalibrating partially parallel acquisition [GRAPPA] = 3; matrix size = 384 × 384; 256 slices). PD images were acquired with the same MPRAGE sequence as used for the collection of the T_1_ weighted images, but without the inversion pulse (TR = 2160 ms; TE = 3.45 ms; flip angle = 4°; GRAPPA = 3; matrix size = 384 × 384; 256 slices; pixel bandwidth = 200 Hz/pixel). A T_2_* dataset was acquired using a modified MPRAGE sequence that allows freely setting the TE (TR = 3700 ms; TE = 16 ms; flip angle = 4°; GRAPPA = 3; matrix size = 384 × 384; 256 slices). Acquisition time for the T_1_, PD, and T_2_* datasets were ~14, 5, and 8 minutes, respectively.

We acquired GE-EPI data during the second scanning session (voxel size = 0.8 mm isotropic; TR = 2400 ms; TE = 22.8 ms; time of volume acquisition [TA] = 1200 ms; silent gap = 1200 ms; GRAPPA = 3; multiband = 2; slices = 36). Slice placement included the bilateral superior temporal plane (STP) and parts of the superior temporal gyrus (STG), covering the auditory cortices. This session comprised 12 runs, each ~5 minutes in duration. A T_1_ weighted scan was acquired for the purpose of realignment across sessions and for slice placement.

### Anatomical data analysis

We divided the T_1_ dataset by the PD images to minimize inhomogeneities induced by the receive coil profile (Van de Moortele *et al*.^57^; left column of Fig. S3). We further corrected the resulting dataset for residual inhomogeneities, down-sampled it to the spatial resolution of the functional data, and brought it to anterior and posterior commissural (ACPC) space. Then, we used BrainVoyager QX 2.8 (Brain Innovation, Maastricht, Netherlands) to identify the WM – GM boundary and the GM – CSF boundary. These boundaries were manually edited to correct segmentation mistakes (i.e., general corrections throughout the brain and detailed editing of both boundaries within the supratemporal plane).

Next, for each subject and hemisphere, we measured cortical thickness using a procedure based on the Laplace equation^52,56,58^ (as implemented in BrainVoyager QX) and defined a cortical depth-dependent grid that covered HG and cortical regions in its immediate vicinity (Figs 3 and S3). These grids contained nine cortical depths ranging from 0.1 (corresponding to deep grey matter) to 0.9 (corresponding to superficial grey matter) cortical depth. The WM-GM and GM-CSF boundaries themselves were not part of the cortical depth-dependent grids.

Myelination is more dense in primary sensory cortices, including in the PAC, than in the surrounding cortex^27,28^, and therefore MRC maps can be used to non-invasively define the PAC in individuals. We created MRC maps by dividing the T_1_ weighted dataset by the T_2_* weighted dataset, which reduced receive coil inhomogeneities and enhanced the intracortical anatomical contrast (see De Martino *et al*.^41^ for details; right column of Fig. S3). Next, the T_1_/T_2_* dataset was corrected for residual inhomogeneities and brought to ACPC space. We limited the MRC maps to the supratemporal plane as segmentations of sufficient precision existed only for this brain region, and created the maps by color coding the intensity of the T_1_/T_2_* dataset within the cortical ribbon. We used the T_2_* weighted dataset to define and remove veins from the MRC maps (see^29^ for details). In each individual hemisphere, the PAC was identified by dividing the cortical depth dependent grid in two parts of equal size based on the MRC map (see Fig. 3 for a representative subject, and Fig. S2 for the data of all subjects). We refer to the half of the grid with higher MRC as PAC, and to the half of the grid with lower MRC as non-PAC.

### Functional data analysis

We analysed the functional data with BrainVoyager QX and custom MATLAB code (The MATHWORKS Inc., Natick, MA, USA). The functional data were pre-processed by correcting for slice scan time, correcting for motion, applying a temporal high pass filter (removing drifts of 2 cycles and less per run), and temporally smoothing the time series (2 data points). We then co-registered the functional to the anatomical data and transformed the dataset to ACPC space.

We estimated the response in each voxel to the natural sounds following the procedure outlined in^37,59^. In short, we first denoised the data^60^ (using the toolbox available at http://kendrickkay.net/GLMdenoise/) and then estimated the hemodynamic response function (HRF) separately for each voxel but common to all sounds^61^. This HRF was used to, per voxel, compute a response estimate (beta weight) for each sound.

Cortical responses to the sounds were analysed with two encoding models that represent different hypotheses on sound processing. A first *frequency* model was simplest, describing sound processing in terms of the frequency preference of cortical neuronal populations. We represented sounds in the space of the *frequency* model by using a biologically-inspired model of auditory cochlear to midbrain processing^62^ (i.e., the first stage of the NSL Tools package; available at http://www.isr.umd.edu/Labs/NSL/Software.htm). That is, the sounds were passed through a filterbank that produces sound spectrograms as output (128 overlapping bandpass filters equally spaced along a logarithmic frequency axis covering 5.3 octaves; minimum frequency = 180 Hz; maximum frequency = 7040 Hz). We averaged these spectrograms over time, which resulted in 128 model parameters to be estimated. A second *spectrotemporal modulation* model described cortical sound processing as the frequency-dependent neuronal processing of spectrotemporal modulations. We represented sounds in the space of the *spectrotemporal modulation* model by passing the output of the first stage of the NSL model to a second model stage that represents cortical auditory processing. That is, a set of modulation filters (temporal modulation rates of ω = [1, 3 9, 27] Hz; spectral modulation scales of Ω = [0.5, 1, 2, 4] cycles/octave) was applied to the sounds’ spectrograms to extract their modulation content. We averaged the resulting sound representation over time, divided the frequency axis in 8 bins with equal bandwidth in octaves, and averaged the modulation energy within each frequency bin, which resulted in 128 model parameters to be estimated (8 frequencies x 4 temporal modulation rates x 4 spectral modulation scales). We refer to the representation of the sounds in the space of the computational models as feature matrix **W** (of size [*S* × *F*], where *S* is the number of natural sounds and *F* is the number of features to estimate).

For each voxel that significantly responded to the training sounds (*p* < 0.05, uncorrected), we estimated the model parameters in 4-fold cross-validation. Each cross-validation used three out of four sound sets for model training (108 training sounds *S*_*train*_), and the remaining sound set was used for model testing (36 testing sounds *S*_*test*_). As these cross-validations followed the sound set division as implemented in the experimental design, in each cross-validation the responses to the training and testing sounds were estimated on completely distinct sets of fMRI data collection runs. We modelled the response to training sounds in each voxel *i* as a linear combination of the features in matrix **W**_**train**_ [*S*_*train*_ × *F*]:

1 $${Y}_{train,i}={{\bf{W}}}_{{\bf{t}}{\bf{r}}{\bf{a}}{\bf{i}}{\bf{n}}}{R}_{i}$$

where *R*_*i*_ [*F*x1] quantifies the contribution of each of the model features to the response of voxel *i*. We used ridge regression^63^ to solve equation 1, where we determined regularization parameter λ for each voxel independently by automatic inspection of the stability of the ridge trace^21,64^. We assessed model performance with a sound identification analysis^21,65,66^, where the model was evaluated by its ability to correctly predict responses to sounds not used in model training (i.e., testing sounds). That is, in each voxel the response to all testing sounds was estimated in the same manner as done for training sounds, with the exception that the voxel-specific HRF was based on the training sounds. Next, we predicted the response to the testing sounds in voxel *i* based on the estimated feature preference *R*_*i*_ [*F*x1] and the representation of the testing sounds in the model space **W**_**test**_ [*S*_*test*_ × *F*]:

2 $${\hat{{Y}}}_{test,i}={{\bf{W}}}_{{\bf{t}}{\bf{e}}{\bf{s}}{\bf{t}}}\,{R}_{i}$$

For each sound *k*, we horizontally concatenated the predicted responses to test sounds *Ŷ*_*test*,*k*_ [1 × *V*] across the four cross-validations, and computed a *sound identification score* by correlating *Ŷ*_*test*,*k*_ to the measured fMRI responses **Y**_**test**_ [*S*_*test*_ × *V*]. For each sound k, we then noted the rank of the correlation between predicted response *Ŷ*_*test*,*k*_ and measured response of test sound *Y*_*test*,*k*_. If the model was able to correctly match the predicted sound *k* with measured sound *k*, this rank would be equal to 1. Instead, a rank equal to *S*_*test*_ represents the worst prediction accuracy, and a rank equal to *S*_*test*_/2 (*S*_*test*_, the number of testing sounds, was equal to 36) represents chance. We defined the prediction accuracy *P*_*k*_ as 1 - the normalized rank:

3 $${P}_{k}=1-\,\frac{{r}_{k}-1}{{S}_{test}-1}$$

Values of *P*_*k*_ range between 0 (i.e., consistently wrong predictions) and 1 (i.e., perfect prediction), with chance = 0.5. We averaged the prediction accuracy across all testing sounds to obtain the model’s overall accuracy. Additionally, we obtained the prediction accuracy per sound category by averaging over the subsets of testing sounds belonging to the same category (i.e. separately for speech, voice, animals, music, tools, and nature sounds). We statistically compared the performance of the models, based on sound responses in the STP as a whole, by Fisher transformation of the prediction accuracy values followed by a two-sided paired t-test.

We assessed the cortical depth-dependent model performance by sampling the responses to the sounds (matrices **Y**) and the trained encoding models (matrices **R**) on the cortical depth-dependent grids and repeating the analysis procedure for deep, middle, and superficial cortical depths. Statistical differences in model performance were assessed separately for the half of the grid with highest and lowest MRC (i.e., the primary and non-primary auditory cortex, respectively). Per MRC-defined dataset, a two-way repeated measures (RM) ANOVA with the factors ‘Computational Model’ and ‘Cortical Depth’ after Fisher transformation of the prediction accuracy values was performed, followed by a multiple comparison corrected paired t-test comparing the performance of the two models per cortical depth.

### Maps of acoustic feature preference

We created maps of feature preference by color-coding each voxel or grid point according to the frequency, temporal modulation rate, or spectral modulation scale with the highest weight in the trained *spectrotemporal modulation* model. The voxels’ best frequency (BF; tonotopy maps) was mapped using a red-yellow-green-blue colour scale, where voxel tuning to low frequencies was indicated in red colours and tuning to high frequencies was indicated in blue. A yellow-green-blue-purple colour scale was used for the maps of temporal modulation rate and spectral modulation scale, where low and high rates and scales were assigned with yellow and purple colours, respectively.

We created group maps by bringing the individual hemispheres in cortex-based aligned (CBA)^67^ space. Each individual map was smoothed (FWHM = 2.4 mm), and sampled in this CBA space. Group maps were computed as the median of the individual subject maps at each vertex that was included in at least 4 individual maps.

## Supplementary information

Supplementary information

## Data Availability

The data will be made available by the corresponding author upon reasonable request.
