# Supplementary material for: Processing complexity increases in superficial layers of human primary auditory cortex
Source: Sci Rep. 2019 Apr 2;9:5502. doi: 10.1038/s41598-019-41965-w (PMC6445291; doi:10.1038/s41598-019-41965-w)
Supplement: Supplementary file 1 — Supplementary information [file 41598_2019_41965_MOESM1_ESM.pdf]

Processing complexity increases in superficial layers of human primary auditory cortex

Supplementary information

Michelle Moerel[a-d], Federico De Martino[b-d], Kâmil Uğurbil[d], Essa Yacoub[d], Elia Formisano[a-c]

[a]Maastricht Centre for Systems Biology, Maastricht University, Universiteitssingel 60, 6229 ER, Maastricht, the Netherlands

[b]Department of Cognitive Neuroscience, Faculty of Psychology and Neuroscience, Maastricht University, Oxfordlaan 55, 6229 EV, Maastricht, the Netherlands

[c]Maastricht Brain Imaging Center (MBIC), Oxfordlaan 55, 6229 EV, Maastricht, the Netherlands

[d]Center for Magnetic Resonance Research, Department of Radiology, University of Minnesota, 2021 6th Street SE, Minneapolis, MN 55455, USA

***Corresponding author:**
Michelle Moerel
Maastricht Centre for Systems Biology, Maastricht University
Universiteitssingel 60
6229 ER, Maastricht
the Netherlands
Phone: +31 43 388 1862
Fax: +31 43 388 4125
michelle.moerel@maastrichtuniversity.nl

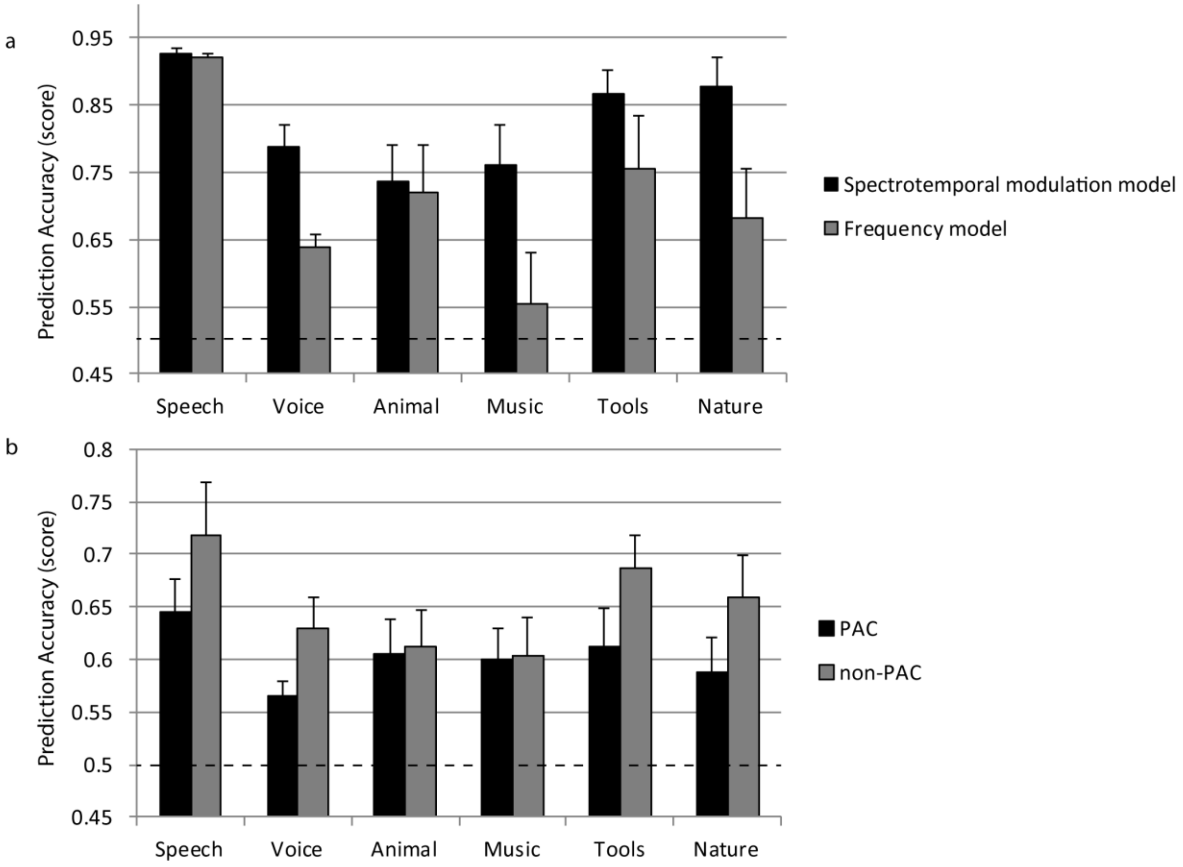

Supplementary Figure 1. Model Performance per sound category.

Model performance separately for each of the six sound categories. (a) Prediction accuracy for each of the models based on the supratemporal plane as a whole. (b) Prediction accuracy for the *spectrotemporal modulation* model, split for the PAC and non-PAC (i.e., the part of the grid with the 50% of highest and lowest myelin-related contrast [MRC], respectively). The dashed line corresponds to chance performance (score = 0.5), and the error bars indicate the standard error across subjects (N = 6) and hemispheres (N = 12) for a and b, respectively.

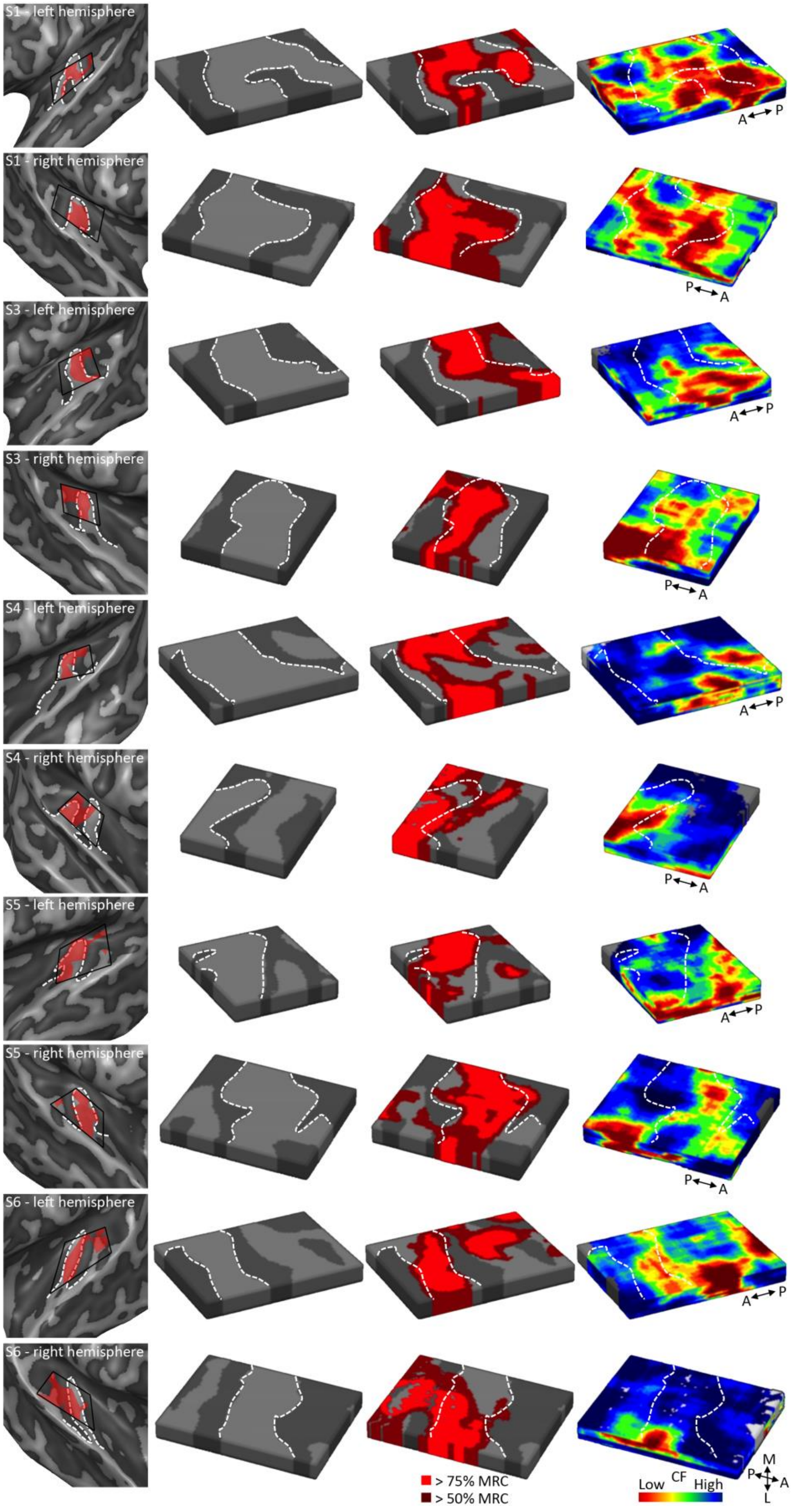
S1 - left hemisphere
S1 - right hemisphere
S3 - left hemisphere
S3 - right hemisphere
S4 - left hemisphere
S4 - right hemisphere
S5 - left hemisphere
S5 - right hemisphere
S6 - left hemisphere
S6 - right hemisphere
A P
P A
> 75% MRC
> 50% MRC
Low CF High
M
P A
L

Supplementary Figure 2. Individual subject data.

Anatomical and functional data of five subjects (see Figure 3 for the data of S2). From left to right, the four columns show (1) the inflated mesh of the WM-GM boundary in the temporal lobe, (2) the cortical depth-dependent grid situated on the postero-medial part of HG, (3) myelin-related contrast (MRC), and (4) the cortical depth dependent tonotopy map. Light and dark grey regions represent gyri and sulci respectively. The white dashed line outlines Heschl's gyrus (HG). The red shaded region in the first column approximates the region with highest MRC (> 50%).

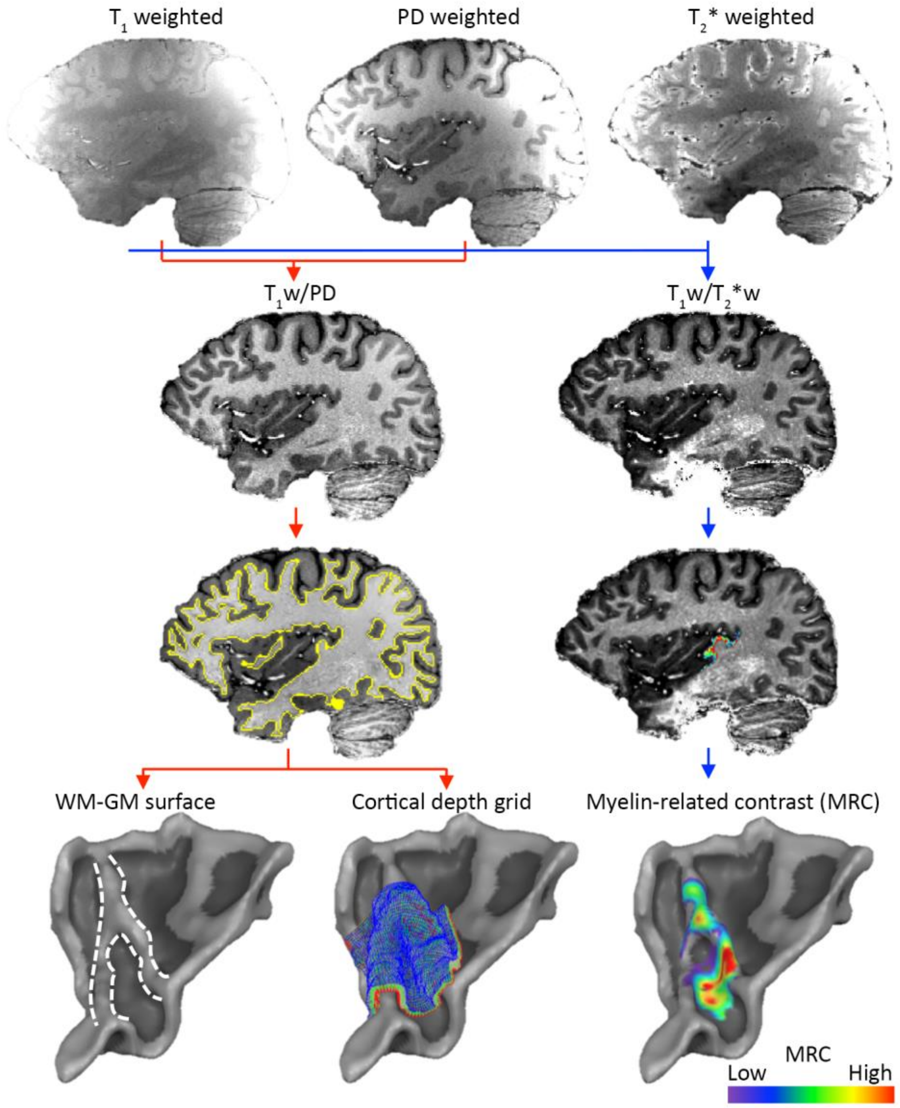

Supplementary Figure 3. Anatomical data analysis.

For each subject, 0.6 mm isotropic T1 weighted (T_1w), proton density weighted (PDw), and T_2* weighted (T_2*w) data are collected. Following the red arrows: the T_1w/PDw image is segmented, and used for the creation of white matter (WM) - grey matter (GM) surfaces and cortical depth dependent grids. Following the blue arrows: the T_1w/T_2*w image is used to create a map of myelin-related contrast (MRC). Anatomical data of S1 are shown.
